# Inner experience differs in rumination and distraction without a change in electromyographical correlates of inner speech

**DOI:** 10.1371/journal.pone.0238920

**Published:** 2020-09-14

**Authors:** Jamie Moffatt, Kaja Julia Mitrenga, Ben Alderson-Day, Peter Moseley, Charles Fernyhough

**Affiliations:** 1 Psychology Department, Durham University, Durham, United Kingdom; 2 School of Psychology, University of Sussex, Brighton, United Kingdom; 3 Department of Psychology, Northumbria University, Newcastle upon Tyne, United Kingdom; Jonkoping University, SWEDEN

## Abstract

Ruminative thought is a style of thinking which involves repetitively focusing upon one’s own negative mood, its causes and its consequences. The negative effects of rumination are well-documented, but comparatively little is known about how rumination is experienced. The evaluative nature of rumination suggests that it could involve more inner speech than non-ruminative states. The present study (*N* = 31) combined facial electromyography and self-report questionnaires to determine the type of inner experience that occurs in rumination. The results showed that induced rumination involved similar levels of muscle activity related to inner speech as periods of induced distraction. However, experience sampling and questionnaire responses showed that rumination involved more verbal thought, and also involved more evaluative and dialogic inner speech than distraction. These findings contribute to the understanding of inner speech as a flexible phenomenon and confirms the importance of employing multiple methods to investigate inner speech. Future research should clarify the link between inner speech in rumination and its negative effects on wellbeing.

## Introduction

Rumination describes a style of thinking that commonly occurs as a maladaptive response to distress and negative mood [1, 2]. Instead of actively engaging with the problems at hand, ruminative thought involves passively and repetitively evaluating one’s own distressing mood, its causes and consequences. Perhaps unsurprisingly, engaging in rumination is closely linked with the onset and maintenance of depression (e.g. [3]) and inducing rumination in participants with depression can directly exacerbate negative mood [4–8]. Furthermore, rumination prompts pessimistic thought patterns [9] and impairs social problem-solving and executive functioning [10–12]. Although a great deal is known about the effects of rumination, comparatively little is known about the underlying mechanisms of the ruminative experience. Understanding the mechanisms of rumination can inform targeted treatments to alleviate the negative effects of ruminative thought. Rumination may consist primarily of “verbal thought”, or inner speech, which can be simply defined as the act of talking to oneself in the mind (for a review, see [13]). Inner speech most frequently consists of self-focused evaluations and thoughts about emotional states [14]. It therefore seems likely that rumination, defined as self-focused, repetitive and negative thoughts, involves more inner speech than non-ruminative states.

The current literature examining the inner experience of rumination has largely employed experience-sampling and interview methodologies. Experience-sampling is a term that describes a diverse range of techniques aimed at capturing experiences in the moment [15]. These approaches have revealed that rumination involves a broad range of processes but may involve more verbal thought than non-ruminative states. In two experiments McLaughlin, Borkovec and Sibrava [16] interrupted participants regularly during a period of induced rumination and asked whether their mental content consisted of primarily verbal-linguistic thought, primarily visual imagery, or something “other”. Across the two experiments, an average of 65.5% of experience samples collected during rumination reported verbal-linguistic thought. A significant proportion of samples collected during rumination reported visual imagery (31.6%), suggesting that rumination is not exclusively a verbal process. Reports of verbal-linguistic thought were equally common during rumination and during a baseline period of unguided thought, but other studies have found significantly more verbal thought during periods of rumination compared to baseline [17, 18]. A limitation of experience sampling methodologies is that they can fail to capture in-depth information about experiences [19]. Experience samples are brief and often require people to report their experience in accordance with narrow, pre-defined criteria. Supporting evidence that rumination involves a diverse range of experiences comes from interviews with people with depression about their ruminative experiences [20]. Of thirty-eight participants, only two reported experiencing exclusively verbal thoughts in rumination. Rumination was experienced “as a feeling” by 76.3% of participants, with visual (52.6%) and auditory (42.1%) experiences also being common. Depressive thoughts, which overlap significantly with ruminative thoughts, involve a range of sensory experiences [21, 22]. Depressed patients describe their depressive cognitions as frequently involving bodily, auditory and visual sensations [22]. In a large non-clinical sample, 38.9% of participants reported having only verbal thoughts in depressive thoughts, whilst a majority reported having visual imagery in depressive thoughts, either with or without accompanying verbal thoughts [21]. A combination of findings from experience-sampling and in-depth reports of rumination therefore strongly suggest that although rumination might involve verbal thought, it is not exclusively verbal. However, it is still unclear if rumination involves more verbal thought than non-ruminative states.

An alternative approach to investigating whether rumination involves inner speech is to examine physiological changes associated with verbal thought. Inner speech is often accompanied by articulatory movements of speech muscles, which can be detected using electromyography (EMG). For example, silent reading is accompanied by elevated levels of activity in the upper (*orbicularis oris superior)* and lower (*orbicularis oris inferior)* lip muscles, compared to rest [23]. This implies that the measurement of articulatory movements can provide a physiological marker of inner speech production. In support of the view that rumination involves inner speech, a recent study found that lip muscles were more active during a period of induced rumination compared to rest, and guided relaxation of those same muscles reduced self-reported rumination [24]. Although this study suggests that rumination involves more lip muscle activity than rest, this may be due to the extra effort required to guide one’s own thoughts. Typically, the effects of induced rumination are compared to a period of induced distraction. Distraction can be thought of as an alternative to rumination as a response to negative mood. It involves directing attention away from negative mood to focus on emotionally-neutral or positive thoughts [2]. Engaging in distraction involves a similar amount of effort to engaging in rumination and therefore provides a more appropriate comparison than a period of rest [25]. Furthermore, naturally occurring rumination can still occur during a period of rest, whereas distraction actively inhibits rumination [11, 25]. The initial aim of the present study was to extend the findings of Nalborczyk et al. [24] by comparing inner speech related muscle activity during induced rumination and induced distraction.

Inner speech is a highly flexible and varied phenomenon, and people frequently report experiencing condensed or fragmentary forms of inner speech in questionnaire surveys [26, 27].These variations may not be easily identifiable when using physiological measures of inner speech. Alderson-Day & Fernyhough [13] argue that much of the time, inner speech could be a basic, condensed representation containing abstract semantic, syntactic and phonological information, which only becomes expanded and more articulated under certain conditions (e.g. in responses to stress or task difficulty). Two key points arise from this theory when considering whether rumination involves more inner speech than non-ruminative states. Firstly, rumination may be expected to prompt a particular kind of expanded inner speech which involves articulation. Secondly, measuring muscle activity is only one way of assessing whether inner speech is taking place during rumination. Investigations need to make use of multiple methods in order to ascertain not only the extent to which rumination involves inner speech, but also what type of inner speech takes places during rumination [28].

In order to determine the extent to which rumination involves inner speech, the present study compared self-reports of inner experience with physiological correlates of articulation during induced rumination and distraction. Experience samples were taken during periods of induced rumination and distraction to determine the extent to which those experiences involved verbal thought and to investigate the type of inner speech that occurs during these two different states. It was expected that rumination would involve more verbal thought than a period of distraction, and that this verbal thought would involve more evaluative and expanded inner speech. After each period, a questionnaire assessed the type of inner speech which took place. Finally, EMG recorded speech-related (OOI, OOS) and emotion-related (*frontalis*, FRO) muscle activity during periods of rumination, distraction and unguided thought. It was expected that rumination would involve more speech and emotion-related muscle activity than distraction and a baseline period of unguided thought. This would suggest that rumination involves more inner speech than non-ruminative states which require similar levels of cognitive effort (distraction), adding to previous findings that rumination involves more physiological correlates of inner speech than periods of rest [24]. Questionnaire measures of trait anxiety, depression and rumination were also included as potential confounding variables.

## Method

### Participants

31 participants (6 males), aged 18–29 (*M* = 20.09, *SD* = 1.78) took part in the study in exchange for course credit. The majority of participants identified as White British (58%) and White Other (19.4%). Ethical approval was given by Department of Psychology Ethics Sub-committee at Durham University. Informed written consent was given by all participants. Participants had no history of psychiatric, developmental or neurological disorder.

### Measures

#### Varieties of inner speech questionnaire revised edition (VISQ-R; [29])

The 26-item questionnaire was used to measure the frequency at which different types of inner speech occur during the preceding thought period. Items are rated on a Likert scale from 1 (“Never”) to 7 (“All the Time”). Factor analyses of the questionnaire have found that each item loads onto one of five varieties of inner speech: condensed/expanded, dialogic, other people, evaluative and positive/regulatory. The present study used an adapted 10-item version of the VISQ-R, with two items taken from each factor. Items with the highest factor loadings in previous studies [29] on each subscale were selected, examples of which are presented in Table 1.

**Table 1:** Examples of items from the VISQ-R for each subscale.

Subscale	Examples
Dialogic	“I was going back and forward asking myself questions and then answering them.”
Evaluative	“In my head, I talked to myself in a critical way.”
Condensed	“My thinking to myself was like shorthand notes, rather than full, proper, grammatical English.”
Positive	“My inner speech helped calm me down.”
Other People	“I experienced the voices of other people asking me questions in my head.”

#### Positive and negative affect scale (PANAS-NA; [30])

The ‘negative affect’ subscale of the PANAS was used to assess negative affect [30]. This is a 10-item questionnaire and each item is rated on a scale from 1 (“Very slightly or not at all”) to 5 (“Extremely”).

#### Rumination and mood scale

Self-report levels of rumination and mood were measured with a series of eight statements. Four of these statements were about different aspects of rumination (e.g. “At this moment I am thinking about my negative things”; adapted from Nalborczyk et al., [24]). The four rumination questions concern the extent to which the participant is thinking is about their feelings, about negative things, about problems and also how self-focused their thoughts are. The remaining four were about positive and negative mood of the participant (e.g. “At this moment, I am happy”; adapted from [31]). Participants were asked to rate how much each statement currently applied to them from 0 to 100, with 0 being “Not at all” and 100 being “Definitely applies to me”.

#### Hospital anxiety and depression scale (HADS; [32])

This 14-item questionnaire measures trait anxiety and depression and consists of seven items related to anxiety (HADS-A) and seven items related to depression (HADS-D). The scale is frequently used to assess both anxiety and depression and shows satisfactory psychometric properties [32].

#### Mini cambridge-exeter repetitive thought scale (mini-CERTS [33])

Trait rumination was measured with this 16-item questionnaire. The mini-CERTS consists of two subscales measuring concrete, experiential thinking and abstract, analytical thinking, otherwise termed as constructive and unconstructive thoughts [33] Items are rated on a Likert scale from 1 (“Almost Never”) to 4 (“Almost Always”).

### Behavioural tasks

#### Negative mood induction

Participants completed two tasks, an anagram task and a subtraction task. Both tasks were designed to be difficult to complete, in order to ensure negative mood induction and have previously been used to induce negative affect as part of a rumination induction procedure [34]. In the anagram task, twenty anagrams were presented on a computer screen for a maximum of thirty seconds each. The anagrams were presented in a fixed order and participants wrote their solutions on an answer sheet provided. If the participant found the solution before that time, or if they found an anagram too difficult, they could skip to the next anagram by pressing a keyboard button. Participants were encouraged to solve as many anagrams as possible in five minutes. Unbeknownst to the participants, six of the anagrams (30%) were unsolvable [35]. None of the participants reached the final anagram in the given time. The anagram task was presented in E-Prime 2.0 (Psychology Software Tools, Pittsburgh, PA). In the subtraction task, participants were asked to count backwards from the number “2083” in steps of thirteen. If an error was made, the experimenter stopped the participant and asked them to start again from “2083”. Participants were encouraged to try to get as far as possible within the five-minute time limit. None of the participants reached 0 in the given time.

#### Rumination and distraction induction

Rumination and distraction were induced by prompting participants to think of a certain topic for six minutes. In the rumination condition, participants were prompted to “Please think about the causes, consequences and meanings of your current feelings”. In the distraction condition, participants were prompted to “Please think about a village, city or town that you are particularly familiar with”. To reduce the possibility of silent reading affecting the EMG recording, the related prompt for each condition was displayed as a single sentence on the screen for five seconds [24]. After the initial prompt, the screen displayed only a fixation cross in the centre of the screen. During each six-minute thought period, participants were interrupted every 85–95 seconds (randomised) for an experience sampling phase, in which they answered five questions presented on the computer screen. At each experience sampling phase, participants rated the extent to which what went through their mind in the previous period could be described as thoughts, images, past-oriented, present-oriented or future-oriented (e.g. “During the previous period, how much of what went through your mind could be described as Thoughts/Images/Past-Oriented/Present-Oriented/Future-Oriented?”) [16, 17]. In the instructions, thoughts were defined as “words that you say to yourself” and images were defined as “pictures in your mind”. Each response was rated on a scale of 0 to 10, with 0 being “Did not occur at all’ and 10 being “Occurred all the time”. Following these questions, participants were again shown the prompt for five seconds, followed by a fixation cross until the next experience sampling phase. Each thought period included four experience sampling phases. The thought induction instructions and probes were presented in E-Prime 2.0 (Psychology Software Tools, Pittsburgh, PA).

### Electromyography

#### Electrode placement & preparation

**Fig 1:**
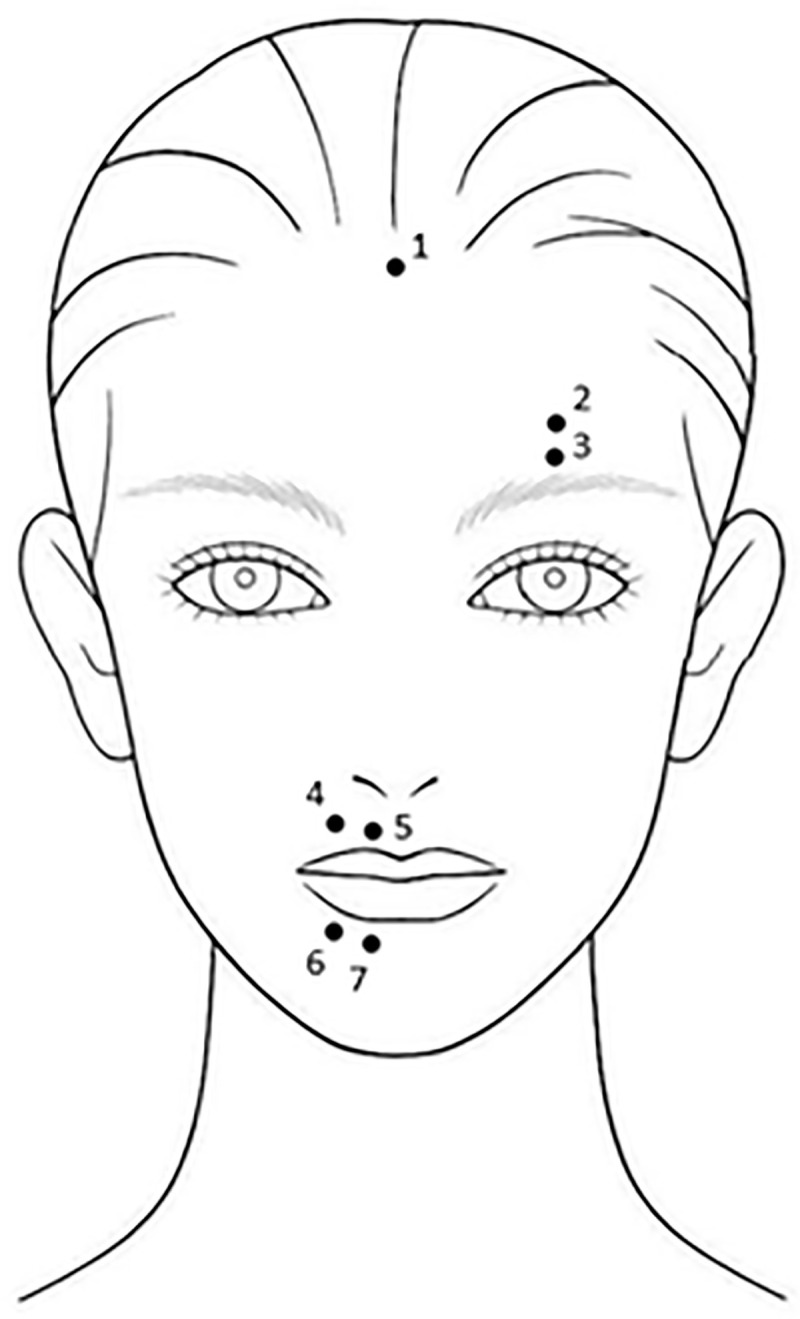
The points indicate where the electrodes were placed for each participant. Electrodes 2–3 recorded signals from the Frontalis muscle. Electrodes 4–5 recorded signals from the orbicularis oris superior muscle. Electrodes 6–7 recorded signals from the orbicularis oris inferior muscle. Electrode 1 was an unshielded grounding electrode.

Muscle activity was recorded from the *orbicularis oris superior* (OOS) and *orbicularis oris inferior* (OOI) lip muscles and the *frontalis* (FRO) brow muscle (as in Nalborczyk et al. [24]). Electrodes were placed according to recommendations on the optimal placement of surface EMG electrodes (see Fig 1). The locations of the OOS and OOI were identified as midway between the facial midline and the corner of the mouth, and as close as possible to the lip vermillion, on the upper lip and lower lip respectively [36]. The location of the FRO was defined as 1cm above the brow, in line with the pupil [37]. Two AgCL electrode discs with a diameter of 4mm were placed either side of each location, 0.5cm apart. Once the electrodes had been attached, participants were asked to make gestures which have previously been shown to elicit activity in the relevant muscles [37, 38]. If these movements failed to produce obvious activity in the EMG signal, the electrode placements were inspected and altered to capture muscle activity more accurately. For safety purposes and to further minimise electrical noise, an unshielded grounding electrode was placed on the upper forehead. To optimise recordings of speech and emotion-related muscle activity, the lip muscle electrodes were placed on the dominant side of the face (e.g. right side for right-handed people), and the brow muscle electrodes were placed on the non-dominant side (e.g. left side for right-handed people), as previous research has shown that emotion-related facial muscle activity is more prominent on the non-dominant side of the face, and speech-related muscle activity on the dominant side of the face [39]. Prior to electrode placement, skin preparation gel (NuPrep Skin Prep Gel, Weaver & Company, Aurora, USA) and 70% isopropyl alcohol were applied to the relevant locations to reduce impedance and ensure optimal signal strength.

#### Data recording

EMG signals were recorded with a surface electromyography system (MP150, BIOPAC Systems, Inc., Goleta, CA, USA). Muscle activity from the FRO, OOS and OOI muscles were amplified by the surface electromyography system with a gain of 2000hz and filtered with a pass band of 10hz to 500hz. Data from each muscle were recorded separately at a sampling rate of 2000 samples per second using AcqKnowledge software (AcqKnowledge 4, BIOPAC Systems, Inc, Goleta, CA, USA). To mark the onset and offset of each thought-induction period, a digital timing pulse was recorded in AcqKnowledge from the E-Prime software (Psychology Software Tools, Pittsburgh, PA, USA) via a parallel port connection. During EMG recording, video and audio recordings of the participant were taken using a webcam (Logitech c270, Logitech, Lausanne, Switzerland) and microphone (Zoom H2n, Zoom Corporation, Tokyo, Japan). Video and audio recordings were inspected later for the removal of obvious movements that were not the target of investigation in this study, according to the guidelines followed by previous research [24].

### Procedure

After consenting to take part, participants completed the HADS, mini-CERTS, PANAS-NA and the Rumination and Mood Scale. The EMG system was set up, and baseline measures of muscle activity were recorded during a period in which participants were asked to remain silent and to not move for one minute. The participants then completed the two negative mood induction tasks, after which participants once again completed the PANAS-NA questionnaire and the Rumination and Mood Scale. Following the negative mood induction, rumination and distraction were induced, the order of which was counterbalanced between participants. After each induction task, participants completed the VISQ-R, as well as the Rumination and Mood Scale.

### Analysis

All questionnaire and experience sampling analyses were conducted using SPSS (IBM SPSS Statistics for Windows, Version 22.0., IBM Corp., Armonk, NY, USA). Unless otherwise reported, the inclusion of order in which the conditions were completed as a between-subjects variable as part of a mixed-design ANOVA produced no significant main effects or interactions involving order.

### Manipulation checks

#### Negative mood induction

To check for the effect of mood induction, PANAS-NA scores and the four ratings of mood were compared pre- and post-mood induction with paired sample t-tests with an adjusted alpha level of 0.01 following a Bonferroni correction for five comparisons. It was expected that negative mood would be increased, and positive mood would be decreased following mood induction.

#### Thought induction

To determine the effect of the two thought induction procedures, ratings of the four rumination statements were compared post-rumination and post-distraction with paired sample t-tests, with an adjusted alpha level of 0.0125 following a Bonferroni correction for four comparisons. It was expected that the induction of rumination would lead to heightened ratings of ruminative thought compared to the distraction induction.

### Self-reported inner experience

#### VISQ-R

Items from each subscale of the VISQ-R (Dialogic, Evaluative, Condensed, Positive, Other People) were summed together, to produce five VISQ-R scores for each condition, for each participant. One participant failed to provide scores for two of the ten VISQ-R Items following the distraction thought period, one item from the Dialogic subscale and one item from the Condensed subscale. The missing values of these items were replaced with the mean item scores derived from the rest of the sample. Subscale scores were compared between conditions using a paired sample t-test, with an adjusted alpha level of 0.01 following a Bonferroni correction for five comparisons. It was expected that rumination would be rated as involving more evaluative, and less condensed inner speech than distraction.

#### Experience samples

The average score across each condition was calculated for each experience sample question (Images, Thoughts, Past-Oriented, Present-Oriented and Future-Oriented). These were compared between rumination and distraction using a paired sample t-test, with an adjusted alpha level of 0.01 following a Bonferroni correction for five comparisons. It was expected that rumination would be rated as involving more thoughts, whereas distraction would involve higher ratings for imagery.

### Electromyography

Three participants were excluded from EMG analyses due to equipment failure, one due to excessive electrical noise, and two due to hardware failures. Data pre-processing was conducted in AcqKnowledge software. EMG data within rumination and distraction were taken from the 20 second periods prior to each experience sample, as marked by the digital signal, and concatenated, resulting in an 80 second period of data for each condition. The decision to use the final 20 seconds of each thought period was made to maximise comparability with previous research investigating muscle activity in rumination, which recorded EMG during the final minute of a five-minute induction period [24]. Baseline recordings involved the full 60 seconds of EMG recording during baseline. Audio and video recordings were recorded in sync with the EMG recordings. The corresponding EMG data of any obvious movement or sound made by the participant were removed based on inspection of the audio and video recordings. Two participants were excluded from further analysis due to excessive movements. In the remaining dataset (n = 26), 8.62% of the data were removed following inspection, and three of the participants had more than 40% of their data removed (41.34%, 51.18% and 50.22%). An FIR filter with Kaiser Bessel windowing was applied to the remaining data, with a band pass filter of 20Hz-450Hz [38].

The pre-processed data were exported to MATLAB (MATLAB r2016a, The MathWorks, Inc., Natick, MA, USA) from Acknowledge. The data were rectified, and the peak amplitude of each muscle (FRO, OOS, OOI) in each condition (Rumination, Distraction and Baseline) for each participant was calculated. Peak values were used as opposed to mean values because the effects of interest were very small, unobservable muscle movements and a mean value would result in these signals being lost amidst the noise of the signal. These peak values were then natural log transformed in order to smooth the data and reduce the impact on the EMG signal of individual differences in muscle movements [40]. All further EMG analyses were conducted in SPSS. A repeated measures ANOVA with condition (Rumination, Distraction, Baseline) as the within-subjects factor was conducted for data from each muscle separately. It was expected that muscle activity in the FRO, OOI and OOS muscles would be heightened in the rumination condition compared to the distraction condition.

## Results

Mean scores for the HADS-A (Mean = 7.13, SD = 3.72) and HADS-D (Mean = 2.81, SD = 2.55) were below the recommended cut-off scores for possible cases of anxiety and depression [41].

### Manipulation checks

#### Negative mood induction

The mean and standard deviations of each mood score pre- and post-mood-induction are presented in Table 2. No significant differences were found for the overall PANAS-NA score (*t*(30) = -0.38, *p* = 0.704, *d* = 0.069). There were also no significant differences in the ratings of the negative mood statements related to ‘upset’ (*t*(30) = -1.74, *p* = 0.092, *d* = 0.312) and ‘sad’ (*t*(30) = -0.85, *p* = 0.402, *d* = 0.153) mood. However, there was a significant reduction in both the ‘happy’ (*t*(30) = 4.23, *p* < 0.001, *d* = 0.76) and ‘optimistic’ (*t*(30) = 3.56, *p* = 0.001, *d* = 0.639) ratings following the negative mood induction.

**Table 2:** Mean mood scores pre- and post-mood induction.

	Pre-Mood Induction	Post-Mood Induction
**PANAS-NA**	14.77 (4.41)	15.06 (3.59)
**Sad**	17.84 (20.41)	20 (18.37)
**Upset**	16.97 (22.34)	23.84 (23.01)
Happy*	64.90 (22.31)	51.97 (21.64)
Optimistic*	60.65 (21.71)	50.01 (21.96)

Standard deviations are reported in brackets.

* indicates that the comparison is significant at the Bonferroni adjusted alpha level (p < 0.01).

#### Self-reported rumination

Table 3 shows means and standard deviations of the ratings of statements related to ruminative thought following each induction. Ratings were significantly higher for Feelings (*t*(30) = 3.165, *p* = 0.004, *d* = 0.568), Negative Things (*t*(30) = 3.776, *p* = 0.001, *d* = 0.678) and Problems (*t*(30) = 4.254, *p* < 0.001, *d* = 0.764) items in the rumination condition, compared to the distraction condition. Ratings of Self-Focus were not significantly different between conditions after controlling for multiple comparisons (*t*(30) = 2.585, *p* = 0.015, *d* = 0.464).

**Table 3:** Mean self-reported rumination scores of rumination and distraction.

	Rumination	Distraction
Feelings*	75.58 (28.74)	61.29 (30.10)
Negative Things*	28.16 (25.61)	13.32 (20.14)
**Self-Focus**	68.45 (23.69)	56.52 (27.89)
Problems*	44.39 (33.10)	24.52 (25.48)

Standard deviations are reported in brackets.

* indicates that the comparison is significant at the Bonferroni adjusted alpha level (p < 0.0125).

Total score on the CERTS scale significantly correlated only with the Problems item during both rumination (*r* = 0.444, *p* = 0.012) and distraction (*r* = 0.518, *p* = 0.03). Scores on the CERTS did not significantly correlate with any other self-reported rumination measure.

### Inner experience

#### Varieties of inner speech

It was expected that rumination would involve more verbal thought than a period of distraction, and that this verbal thought would involve more evaluative and expanded inner speech. Table 4 shows means and standard deviations of the ratings of varieties of inner speech (VISQ-R) in rumination and distraction. Ratings of Dialogic and Evaluative inner speech were significantly higher in the rumination condition than in the distraction condition (*t*(30) = 5.977, *p* < 0.001 *d* = 1.073; *t*(30) = 4.509, *p* < 0.001, *d* = 0.81). No other subscales showed a significant difference (all *t*(30) < 1.5, all *p* > 0.14, all *d* < 0.266).

**Table 4:** Mean scores on the varieties of inner speech questionnaire.

	Rumination	Distraction
Dialogic*	9.03 (2.24)	6.69 (2.39)
Evaluative*	7.39 (2.25)	5.35 (2.43)
**Condensed**	5.94 (3.32)	6.93 (3.39)
**Positive**	8.26 (2.46)	8.03 (2.15)
**Other People**	3.32 (2.10)	3.23 (2.32)

Standard deviations are reported in brackets.

* indicates that the comparison is significant at the Bonferroni adjusted alpha level (p < 0.01).

To determine if the order of the two inductions influenced inner speech, order was included as a between-subjects variable as part of a mixed-design ANOVA for each of the VISQ subscales. Although there were no significant main effects for order or induction type for Condensed (F(1, 29) = 2.79, p = .106, $\mu_{p}^{2}$ = .088; F(1, 29) = 3.055,p = .091, $\mu_{p}^{2}$ = .095) or Positive (F(1, 29) = 2.53, p = .122, $\mu_{p}^{2}$ = .08; F(1, 29) = .186, p = .669, $\mu_{p}^{2}$ = .006) inner speech, there were significant interactions for both subscales (*F*(1, 29) = 8.37, *p* = .007, $\mu_{p}^{2}$ = .224; *F*(1, 29) = 4.90, *p* = .035, $\mu_{p}^{2}$ = .145). Further analyses were conducted to clarify these significant interactions, though it should be noted that some argue that significant interactions without corresponding significant main effects are implausible and should be interpreted with caution [42]. Independent t-tests revealed that inner speech in the rumination condition was rated as more Condensed if it was engaged in first, compared to if it was completed second (*t*(29) = 3.237, *p* = 0.003, *d* = 1.163). The rumination condition was rated as involving more Positive inner speech by those who engaged in rumination first, compared to those who completed rumination second (*t*(29) = 2.513, *p* = 0.018, *d* = 0.903). In summary, rumination tended to involve more Condensed and Positive inner speech if engaged in first.

#### Experience sampling

Experience sampling was used to investigate the nature of inner experience during periods of rumination and distraction. Means and standard deviations for experience sample scores in rumination and distraction can be seen in Table 5. Rumination produced significantly higher ratings than distraction in Thoughts (*t*(30) = -6.17, *p* < 0.001, *d* = 1.108), Present-Oriented (*t*(30) = -5.097, *p* < 0.001, *d* = 0.916) and Future-Oriented (*t*(30) = -5.11, *p* < 0.001, *d* = 0.917) experience samples. Distraction produced significantly higher ratings than rumination on Images (*t*(30) = 9.95, *p* < 0.001, *d* = 1.787) and Past-Oriented (*t*(30) = 7.72, *p* < 0.001, *d* = 1.387) experience samples.

**Table 5:** Mean scores on experience samples.

	Rumination	Distraction
Images*	3.82 (2.20)	7.41 (2.00)
Thoughts*	7.95 (1.41)	6.02 (2.00)
Past-Oriented*	4.05 (1.92)	7.02 (1.59)
Present-Oriented*	6.71 (4.12)	2.80 (1.52)
Future-Oriented*	5.29 (1.95)	3.06 (2.28)

Standard deviations are in brackets.

* indicates that the comparison is significant at the Bonferroni adjusted alpha level (p < 0.01).

### Electromyography

**Fig 2:**
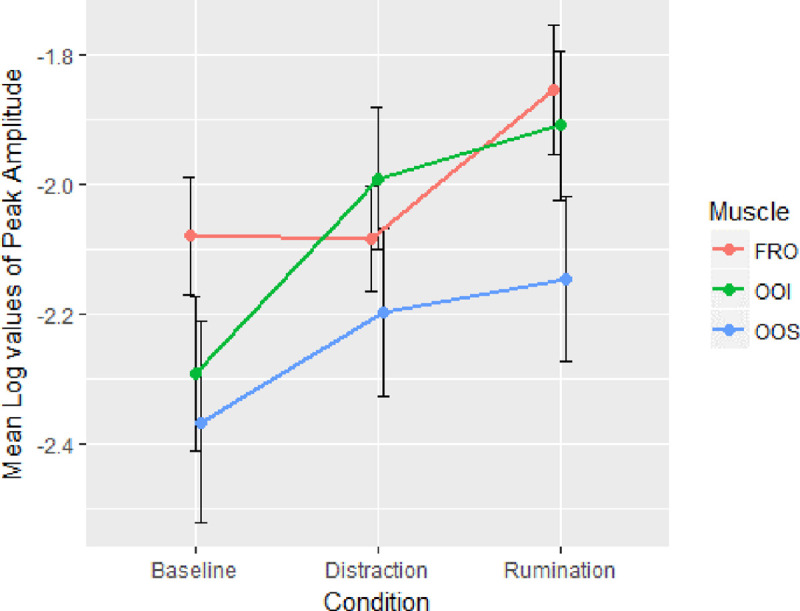
The average of the log-transformed peak amplitudes of the frontalis (FRO), orbicularis oris inferior (OOI) and orbicularis oris superior (OOS) muscles, in each of the experimental conditions (Baseline, Distraction, Rumination). A higher value indicates heightened muscle activity. The FRO showed significantly heightened muscle activity in rumination compared to distraction and baseline. The OOI muscle showed significantly heightened muscle activity in rumination and distraction compared to baseline. The OOS muscle showed no significant difference between the conditions. Error bars are standard errors from the mean.

Rumination was expected to involve more speech and emotion-related muscle activity than distraction and a baseline period of unguided thought. The mean values of the log-transformed peak amplitudes for each muscle in each condition can be seen in Fig 1. Three one-way ANOVAs were carried out to investigate the difference in muscle activity between the FRO, OOI and OOS muscle activity in the rumination, distraction and baseline conditions. The mean values of the log-transformed peak amplitudes for each muscle in each condition can be seen in Fig 2. For FRO muscle activity, the assumption of sphericity was violated and Greenhouse-Geisser values are therefore reported. A significant main effect of condition (*F*(1.531, 38.227) = 8.487, p = 0.002, $\mu_{p}^{2}$ = 0.253) was found. Post-hoc analysis with Bonferroni correction applied revealed a significant difference in FRO muscle activity between rumination and distraction (*p* = .006) and between rumination and baseline (*p* = .020). There was no significant difference between distraction and baseline (*p*>.05). This suggests that the FRO muscle was more active during the rumination condition compared to both the distraction and baseline conditions.

The mean peak log values for the OOS muscle can be seen in Fig 1. Muscle activity from the OOS showed no significant main effect of condition (*F*(2, 50) = 2.395, *p* = 0.102, $\mu_{p}^{2}$ = 0.087). The mean peak log values for the OOI muscle can be seen in Fig 1. Muscle activity from the OOI showed a significant main effect of condition (*F*(2, 50) = 6.258, *p* = 0.004, $\mu_{p}^{2}$ = 0.201). Post-hoc tests with Bonferroni correction applied revealed a significant difference between rumination and baseline conditions (*p* = .013) and distraction and baseline conditions (*p* = .049). No significant difference in OOI muscle activity was found between rumination and distraction (*p*>.05). This suggests that the OOI muscle was more active during the rumination and distraction conditions than during the baseline condition.

It was expected that lip muscles would show increased activity during the period of induced rumination compared to distraction. The findings instead supported the null hypothesis that rumination and distraction involve similar levels of lip muscle activity. One possible explanation is that the study is underpowered. The average effect size previously reported for comparing muscle activity between a period of rumination and rest is *d* = 0.72 [24]. A power analysis conducted with an equivalent f-test effect size of *f* = 0.36, with 80% power and an alpha of 0.05, estimated a sample size of 40, therefore it is possible that the sample size of the present study lacked sufficient power to detect the effect of rumination on muscle activity. In order to test this, a Bayesian paired samples t-test was conducted for the peak log values of muscle activity between the rumination and distraction conditions. This revealed strong evidence in favour of the alternative hypothesis for the FRO muscle (*B*_10_ = 18.79), and moderate evidence in favour of the null hypothesis for the OOS (*B*_10_ = 0.232) and OOI (*B*_10_ = 0.278) muscles, according to current guidelines for interpreting Bayes factors [43].

## Discussion

The initial aim of the present study was to extend the findings of Nalborczyk et al. [24], by investigating the differences in muscle activity between induced rumination and induced distraction. A second and broader aim of the study was to compare self-reported measures and physiological correlates of inner speech during induced rumination, to determine what type of inner experience takes place in rumination.

Comparisons of peak muscle activations revealed that the brow muscle *frontalis* was significantly more active during a period of rumination, compared to distraction and a baseline period of rest. The lower lip muscle *orbicularis oris inferior* was significantly more active during rumination compared to baseline, whereas the upper lip muscle *orbicularis oris superior* showed no significant difference in activity between the three conditions. The *frontalis* muscle is associated with negative mood [37], and was therefore expected to show heightened activity during rumination. As in Nalborczyk et al. [24], the present study found that lower lip muscles are more active during rumination compared to rest but extends this research by finding that both lower and upper lip muscles showed no more activation during rumination when compared to a period of distraction. Distraction was included as a control condition, in addition to a baseline condition of rest, to determine if elevated lip muscle activity is specific to rumination, or if it is due to the effort required to guide one’s own thoughts. Given that lip muscle activity has previously been related to inner speech production [23], this suggests that rumination has no specific boost of inner speech production relative to distraction. The increase in lip-muscle activity during states where thoughts were guided (rumination, distraction), compared to rest may therefore reflect the extra effort required to guide one’s thoughts and to engage in inner speech production.

Whilst the EMG findings appear to suggest that rumination and distraction involve inner speech to a similar degree, self-report measures revealed striking differences in how each thought period was experienced. Experience sampling found that participants reported significantly more verbal thought during rumination than distraction, and significantly more imagery during distraction than rumination. This is in line with previous research which demonstrated that rumination is a primarily verbal phenomenon [17]. Furthermore, a questionnaire assessing the variety of inner speech showed that rumination involved more evaluative and dialogic inner speech than distraction. Ruminative thinking is frequently defined as an evaluative mode of thought [2], but no previous study has suggested that rumination should preferentially engage dialogic inner speech. An order effect was found for the condensed and positive subscales of the VISQ-R. Rumination involved less condensed inner speech when engaged in after the distraction condition, and more positive inner speech when engaged in before the distraction condition. Lower scores on the condensed inner speech subscale of the VISQ-R can be interpreted as indexing higher levels of expanded inner speech, since these forms of inner speech are conceived of as representing poles on a continuum [29, 44]. Our findings suggest that rumination involves an expanded form of inner speech, but only when following a period of distraction.

It is noteworthy that although a physiological correlate of inner speech evidenced no differences between rumination and distraction, there were differences in self-reported inner speech between these two conditions. This suggests that the measurement of articulatory movements failed to adequately capture the variety in the experience of inner speech. There are several reasons for caution when using articulatory movements alone as a marker for inner speech. Firstly, previous studies have demonstrated that inner speech does not necessarily require motor activity, implying that at least some inner speech cannot be detected with EMG. Individuals who are temporarily unable to produce speech-related movements, either through muscle-paralysing injections [45] or neurostimulation [46] still report experiencing inner speech. Secondly, inner speech can differ in form depending on the extent to which articulatory muscles are involved. Increasing the involvement of articulatory muscles by silently mouthing during silent speech generation appears to affect the phonological detail of inner speech representations [47]. This suggests that only certain types of inner speech, such as more phonologically rich inner speech, may be detectable by EMG. This underlines that inner speech is a highly variable phenomenon [12]. Future studies should ensure that any putative physiological markers of inner speech, such as lip muscle activity, are accompanied by questionnaire methods which are sensitive to the phenomenological variability of inner speech. It has previously been argued that multiple methods are necessary to comprehensively assess inner speech [28]. The use of multiple methods in the present study allowed us to demonstrate that rumination may involve a similar amount of inner speech to non-ruminative thought, but with key differences in how it is experienced.

It was expected that rumination would involve more evaluative inner speech than distraction, given that is often defined as an evaluative mode of thought [2]. Rumination was also rated as involving more dialogic inner speech than distraction. The Dialogic subscale of the VISQ-R measures the extent to which inner speech resembles a conversation with either oneself or another person in the mind [26, 29]. In a recent, revised version of the VISQ, scores on the Dialogic subscale correlated with all other subscales, particularly Evaluative inner speech [29]. This led the authors to claim that dialogic inner speech might represent a core feature of inner speech, in accordance with Vygotskian theories of inner speech. Rumination in depressed individuals most commonly consists of thoughts about relationships with other people, and about how past events might have played out differently [20]. Previous research suggests that these topics might preferentially engage dialogic inner speech. Firstly, dialogic inner speech has been shown to activate the same brain regions associated with representing the minds of others [48], and might therefore be particularly useful for social cognition. Secondly, creative thoughts [49] and moral reasoning [50] are also thought to be closely related to dialogic inner speech, suggesting that re-imagining previous scenarios and subsequent consequences might engage dialogic thinking.

A key limitation of the present study is that the negative mood induction did not produce an increase in negative mood, although it did significantly decrease positive mood. Inducing rumination in non-clinical participants is most commonly achieved by inducing negative mood before presenting prompts to ruminate. Indeed, the tasks used in the present study were chosen because they have previously been used to induce negative mood as part of a rumination induction [34]. Many of the maladaptive effects of rumination only occur in people who are experiencing negative emotions prior to the prompt to ruminate [25]. For example, inducing rumination in depressed patients exacerbates negative mood relative to distraction, but no such effect is found in non-depressed controls [5]. These findings suggest that rumination occurring during negative mood is very different from rumination occurring during neutral or positive emotional states, and this might be reflected in how it is experienced. In the present study, self-report measures indicated that participants ruminated more following the rumination induction than following the distraction induction, despite the limited impact of the mood induction. Moreover, the experience sampling analyses revealed that rumination consisted of similar levels of verbal thought and visual imagery to those reported in previous studies [16, 17]. It therefore seems likely that the lack of negative mood did not impact the efficacy of the rumination induction. A promising avenue of future research would be to further investigate the experience of rumination when it arises from negative mood compared to positive or neutral mood.

Another aim for future research should be to determine whether the different types of inner speech employed in rumination and distraction can account for their differing impact on general well-being. Rumination is considered to be a maladaptive response to negative mood, whereas distraction is an alternative adaptive response [2]. Two aspects of the experience of rumination reported in the present study may contribute to the detrimental impact of rumination on well-being. Firstly, rumination involved more experiences of verbal thought than distraction. The use of verbal thought has previously been closely linked with negative mood. People encouraged to think about hypothetical scenarios with verbal thought showed reduced mood [51], and were more susceptible to a subsequent negative mood induction [52] than those encouraged to think visually. Secondly, rumination employed more evaluative inner speech than distraction. Evaluative inner speech is negatively associated with self-esteem [44]. The results of the present study suggest that excessive use of inner speech, particularly evaluative inner speech, may be an underlying mechanism which leads to rumination, and subsequently its negative effects. Future research should directly investigate the link between inner speech in rumination and well-being, and test targeted interventions for alleviating the negative effects of rumination by influencing inner speech.

In conclusion, induced rumination appeared to involve similar levels of inner speech-related muscle activity to a period of distraction. However, self-reports revealed a considerable difference in how ruminative and non-ruminative thoughts are experienced. Rumination consisted of more verbal thoughts, as well as dialogic and evaluative inner speech, compared to a period of distraction. The combined use of multiple methods revealed nuances to inner speech in rumination that would have been missed by using either self-reports or EMG alone [29]. Specifically, this novel and comprehensive approach revealed that ruminative thinking involves different kinds of inner speech to non-ruminative thought, but these different kinds of inner speech are not clearly associated with speech-related muscle activity.
